# A novel proteomic-based model for predicting colorectal cancer with *Schistosoma japonicum* co‐infection by integrated bioinformatics analysis and machine learning

**DOI:** 10.1186/s12920-023-01711-8

**Published:** 2023-10-30

**Authors:** Shan Li, Xuguang Sun, Ting Li, Yanqing Shi, Binjie Xu, Yuyong Deng, Sifan Wang

**Affiliations:** 1 Precision Preventive Medicine Laboratory of Basic Medical School, Jiujiang University, Jiujiang, 332005 China; 2 Art School, Jiujiang University, Jiujiang, 332005 China; 3 Affiliated Hospital of Jiujiang University, Jiujiang, 332005 China

**Keywords:** *Schistosoma Japonicum*, Differentially expressed genes, Machine learning, Diagnostic values

## Abstract

*Schistosoma japonicum* infection is an important public health problem and the *S. japonicum* infection is associated with a variety of diseases, including colorectal cancer. We collected the paraffin samples of CRC patients with or without *S. japonicum* infection according to standard procedures. Data-Independent Acquisition was used to identify differentially expressed proteins (DEPs), protein–protein interaction (PPI) network construction, Gene Ontology (GO) and Kyoto Encyclopedia of Genes and Genomes (KEGG) functional enrichment analysis and machine learning algorithms (least absolute shrinkage and selection operator (LASSO) regression) were used to identify candidate genes for diagnosing CRC with *S. japonicum* infection. To assess the diagnostic value, the nomogram and receiver operating characteristic (ROC) curve were developed. A total of 115 DEPs were screened, the DEPs that were discovered were mostly related with biological process in generation of precursor metabolites and energy,energy derivation by oxidation of organic compounds, carboxylic acid metabolic process, oxoacid metabolic process, cellular respiration aerobic respiration according to the analyses. Enrichment analysis showed that these compounds might regulate oxidoreductase activity, transporter activity, transmembrane transporter activity, ion transmembrane transporter activity and inorganic molecular entity transmembrane transporter activity. Following the development of PPI network and LASSO, 13 genes (hsd17b4, h2ac4, hla-c, pc, epx, rpia, tor1aip1, mindy1, dpysl5, nucks1, cnot2, ndufa13 and dnm3) were filtered, and 3 candidate hub genes were chosen for nomogram building and diagnostic value evaluation after machine learning. The nomogram and all 3 candidate hub genes (hsd17b4, rpia and cnot2) had high diagnostic values (area under the curve is 0.9556). The results of our study indicate that the combination of hsd17b4, rpia, and cnot2 may become a predictive model for the occurrence of CRC in combination with *S. japonicum* infection. This study also provides new clues for the mechanism research of *S. japonicum* infection and CRC.

## Author summary

Colorectal cancer is the third most common malignant tumor globally, but the exact pathogenic causes and related mechanisms still require further research. Investigations have shown that about 17% of tumor patients can be attributed to chronic infections, and infection is considered one of the main factors in tumor development. Previous studies have indicated that the incidence of colorectal cancer in patients infected with *Schistosoma japonicum* is significantly higher than that of the normal population, with a three-fold higher risk of developing the disease than non-infected individuals. However, the exact mechanism of their interaction is not yet entirely clear. Our study constructed a predictive model for *S. japonicum*-related colorectal cancer through the collection of clinical data and proteomic analysis, providing scientific guidance for clinical diagnosis and precision treatment. Secondly, it provides a theoretical basis for the study of the relationship between *S. japonicum* infection and colorectal cancer.

## Introduction

The worldwide prevalence of colorectal cancer (CRC) has been steadily increasing due to population growth. CRC ranks as the third most frequently diagnosed cancer and is the second leading contributor to cancer-related deaths [1]. Multiple genetic and environmental factors have been implicated in colorectal carcinogenesis, including inflammatory bowel disease [3]. Investigation shows that about 17% of tumor patients can be attributed to chronic infection, infection is considered to be one of the main factors of tumor development [5].

Schistosomiasis, caused by blood-dwelling flukes, is one of the most prevalent parasitic diseases, which affecting multiple organs, notably the intestines, liver, and bladder. Five schistosome species are known to cause human infection: *Schistosoma haematobium*, *Schistosoma mansoni*, *Schistosoma mekongi*, *Schistosoma intercalatum*, and *Schistosoma japonicum*. *S. haematobium* infection is one of the 11 infectious pathogens classified as Group I carcinogens by The International Agency for Research on Cancer (IARC) [6]. However, *S. japonicum* is the only Schistosoma species responsible for human infection in China, particularly in 12 provinces south of the Yangtze River [8]. Although China has achieved great progress in controlling *S. japonicum* infection, but it remains a public health problem fou our health. *S. japonicum* has been classified by the IARC as a probable carcinogen in humans (class 2B) causing liver cancer. A growing body of epidemiological and pathological evidence is implicating *S. japonicum* infection in colorectal carcinogenesis, leading to tumors with a distinct biological behavior. Nonetheless, the experimental evidence supporting this association is meager, and few studies have comprehensive compared the discriminatory capacity of noninvasive markers with *S. japonicum* infection. Therefore, identification of valuable biomarkers or establishment of a model that can predict the incidence of CRC after *S. japonicu* infection are especially important. It is also of great significance for our study of the relationship between schistosomiasis infection and colorectal cancer.

## Results

### Patient characteristics

A total of 15 patients were successfully paired for subsequent detection and analysis. The aim of this study is to predict the prognosis, and therefore participants were grouped according to their prognosis results, each colorectal cancer patients with or without *S. japonicum* infection were matched in gender, age, T, N, M staging and surgical status and other clinical indicators. Information of patient characteristics was shown in Table 1.

**Table 1:** The basic information of the patients

		colorectal cancer patients	
		without *S. japonicum* infection	with *S. japonicum* infection
Total number (N)		15	15
Gender (n,%)			
	Female	2 (13.33)	3 (20.00)
	Male	13 (86.67)	12 (80.00)
Age, mean ± SD		72.7 ± 4.9	72.3 ± 4.5
T Stage(%)	1	2 (13.33)	1 (6.67)
	2	1 (6.67)	2 (13.33)
	3	12 (80.00)	12 (80.00)
N Stage (%)	0	7 (46.67)	8 (53.33)
	1	7 (46.67)	6 (40.00)
	2	1 (6.67)	0 (0.00)
	3	0 (0.00)	1 (6.67)
M Stage (%)	5	15 (100.00)	15 (100.00)
Stage (%)	2	15 (100.00)	15 (100.00)
surgery (%)	1	15 (100.00)	15 (100.00)

Stage 2: Medium-low, medium-high and medium-high differentiation. All patients underwent surgery, defined as 1

### Proteomic analysis of paraffin sections

**Fig. 1:**
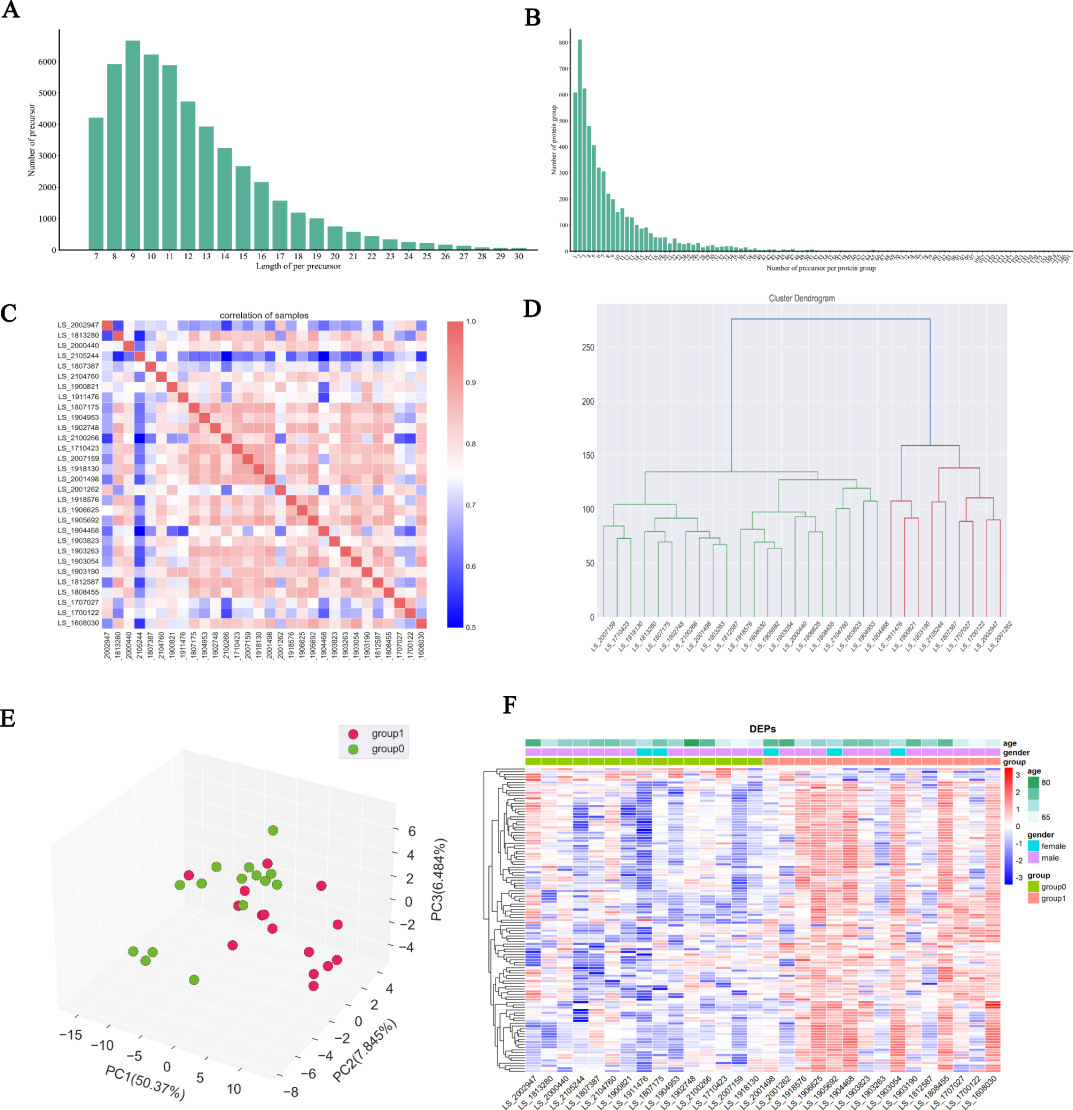
Quality control in proteomics experiments. (**A**) The length distribution of most peptides detected by mass spectrometry falls within 7–27 amino acids. (**B**) Over 99% of proteins are multi-peptide proteins. (**C**) Pearson correlation analysis shows good reproducibility among samples. (**D**) Hierarchical clustering analysis of samples. (**E**) Using hierarchical clustering analysis, individual samples can be classified into different population groups. (**F**) The quantitative heatmap of differentially expressed proteins

We identified a total of 5,614 proteins and 62,225 peptides in this study. To ensure the quality of our experimental results, we plotted the distribution of peptide lengths and numbers for each group, as shown in Fig. 1. The mass-to-charge ratio (m/z) range for MS1 scans is typically 350–1500, and the majority of peptide ions have a charge of + 2 (with decreasing charge as + 3, +4, etc.). The average molecular weight of amino acid residues in proteins is approximately 110 Da, and most peptides contain 7–27 amino acids (Fig. 1A) and 99% of the proteins are multi-peptide proteins (Fig. 1B). Based on these data, we can conclude that the sample preparation met the basic requirements for proteomics analysis, and the results obtained from subsequent experiments are convincing. Principal Component Analysis (PCA) analysis was used to evaluate the quantitative reproducibility among samples and the quantitative relationships among different sample groups (Fig. 1C-E). The results indicate good sample reproducibility, which can be used to evaluate and screen for samples with quantitative abnormalities in this group. A heatmap was generated using hierarchical clustering to display the quantitative information (Fig. 1F), which reveals significant protein differences among samples from the colon cancer group and the colon cancer combined with *S. japonicum* infection group. These differentially expressed proteins appear to serve as a basis for differential diagnosis and can provide insights into the role of *S. japonicum* infection in the occurrence and development of colon cancer.

### Differentially expressed proteins screening and functional enrichment analysis

**Fig. 2:**
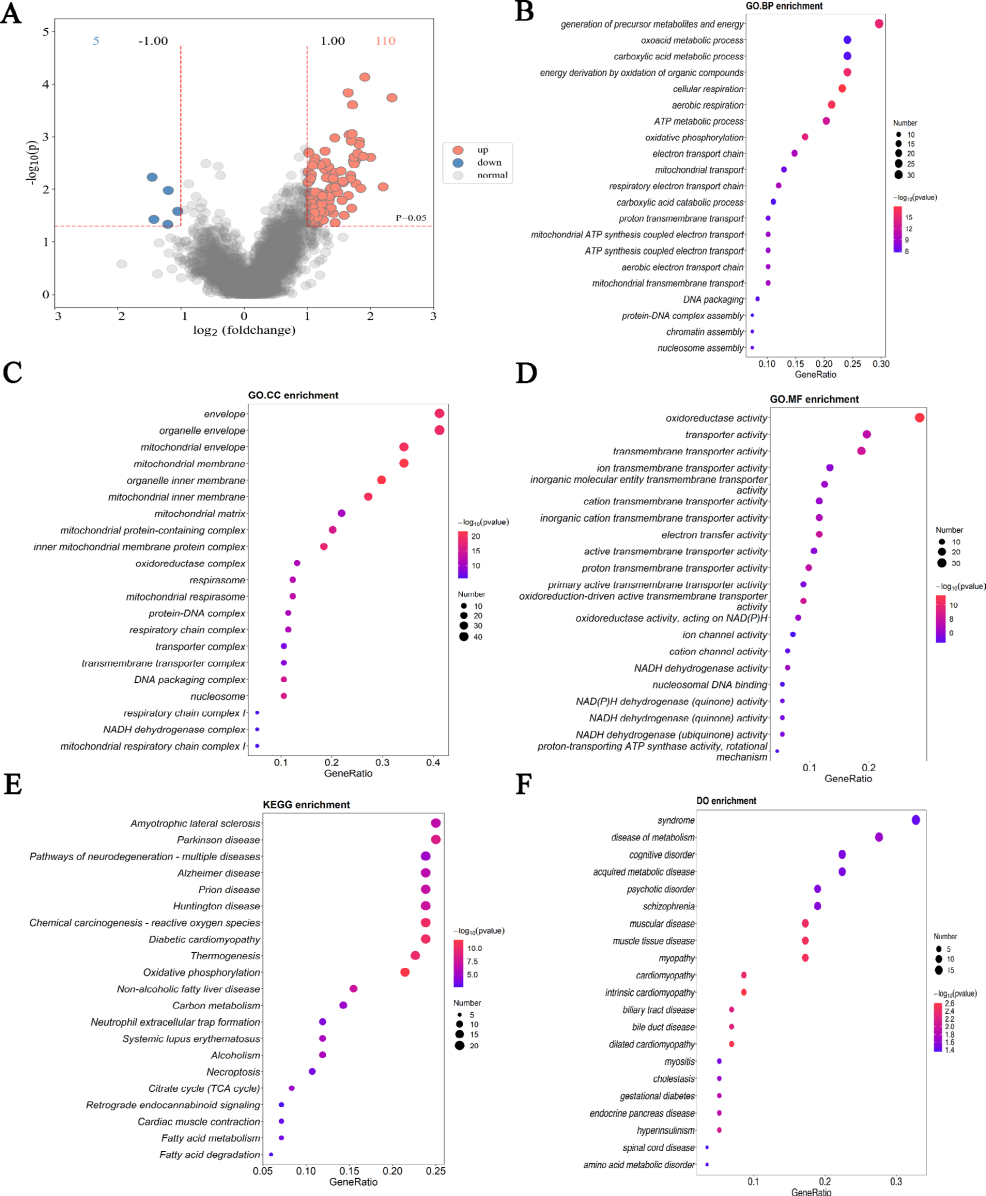
The differentially expressed proteins (DEPs) screening and annotation enrichment analysis. (**A**) Volcano map of DEGs, a total of 115 DEPs were screened out, including 110 up-regulated proteins and 5 down-regulated proteins. The red represents up-regulated differential genes, green represents down-regulated differential genes and black represents no significant difference genes. (**B**-**D**) Gene Ontology (GO) enrichment analyses of DEGs, including biological process (BP), cellular component (CC), and molecular function (MF), respectively. The y-axis represents different GO terms, the x-axis represents gene ratio enriched in relative GO terms, the circle size refers to gene numbers, and the color represents -log10 (p-value). (**E**) Kyoto Encyclopedia of Genes and Genomes (KEGG) enrichment analyses of DEGs, the Fig shows the top 20 enriched pathways. (**F**) Disease Ontology (DO) enrichment analysis was performed on DEGs and the top 20 terms were selected for visualization

Compared with the pure colorectal cancer samples, a total of 115 differentially expressed proteins were screened, including 110 up-regulated proteins and 5 down-regulated proteins, which was presented in the volcano plot (Fig. 2A). The Gene Ontology (GO) and Kyoto Encyclopedia of Genes and Genomes (KEGG) enrichment analysis were performed for the DEPs that were screened and the top 20 enriched terms are presented in Fig. As shown in Fig. 2B, the DEGs were enriched in biological process including the generation of precursor metabolites and energy, oxoacid metabolic process, carboxylic acid metabolic process, energy derivation by oxidation of organic compounds, cellular respiration aerobic respiration. The top five annotation information of differential proteins enriched in cell components were organelle envelope, mitochondrial membrane, mitochondrial envelope, organelle inner membrane, mitochondrial inner membrane (Fig. 2C). The DEGs were enriched in oxidoreductase activity, transporter activity, transmembrane transporter activity, ion transmembrane transporter activity, inorganic molecular entity transmembrane transporter for molecular function (Fig. 2D). Otherwise, the KEGG pathway was enriched for parkinson disease, amyotrophic lateral sclerosis, diabetic cardiomyopathy, chemical carcinogenesis-reactive oxygen species, huntington disease, neutrophil extracellular trap formation, necroptosis (Fig. 2E). The findings of the DO study are depicted in Fig. 2F, and the most common diseases enhanced by DEGs were syndrome, disease of metabolism, acquired metabolic disease.

**Fig. 3:**
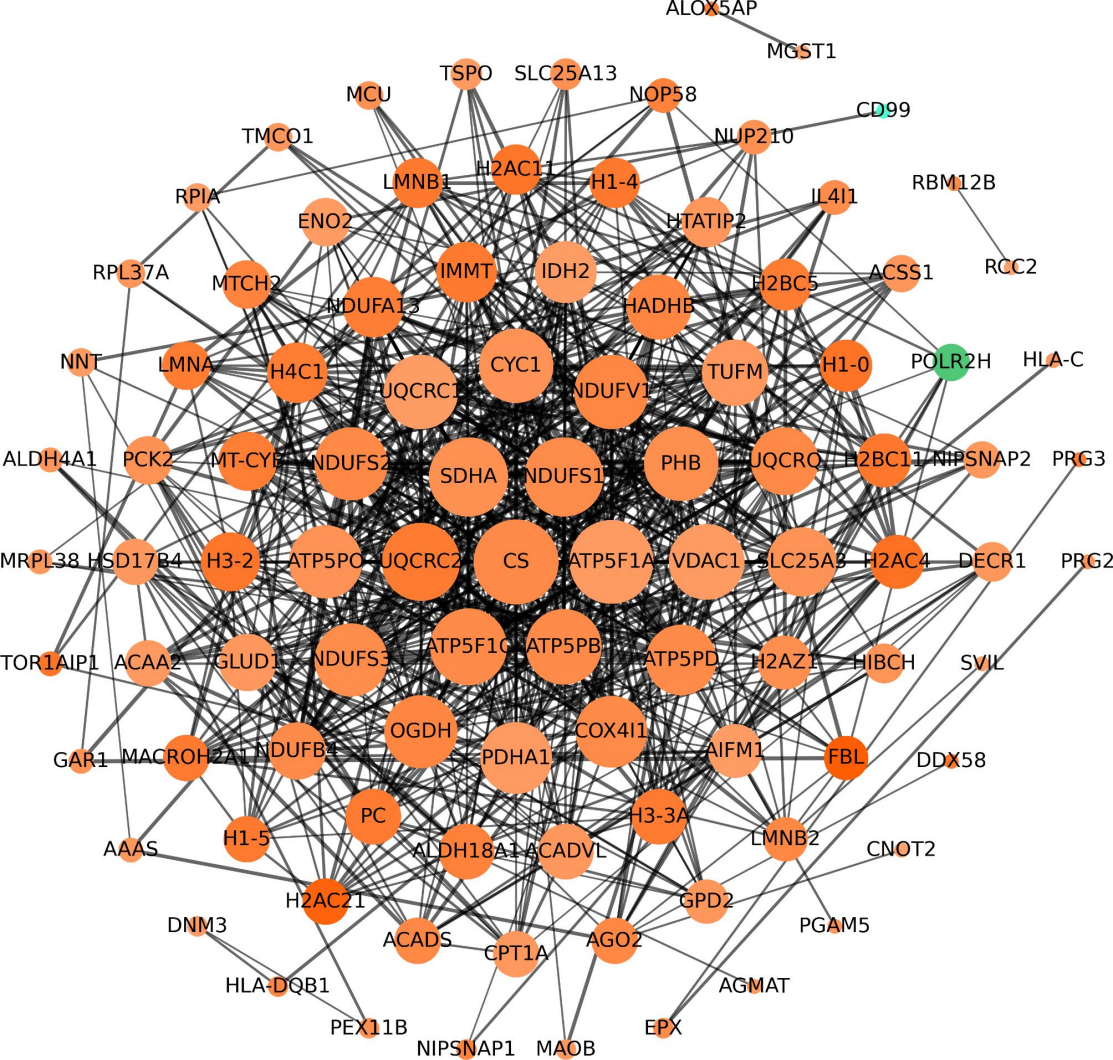
Protein-protein interaction (PPI) network analysis of DEPs. The minimum required interaction score was 0.4, green indicates downregulated proteins, while orange indicates upregulated proteins. The lines between the nodes represent their interactions, and the thickness of the lines represents the interaction score, which is theoretically related to the strength of the interaction. Nodes that are closer to the center indicate that they interact with more proteins. DEPs, differentially expressed proteins

The STRING database is a data system used to retrieve known and predicted protein-protein interactions, and contains extensive protein information. It is currently the most widely used and comprehensive protein interaction network analysis tool. In our study, the protein-protein interaction networks of differential proteins were analyzed and compared based on the STRING database, the results as shown in Fig. 3. The top 10 highdegree core DEPs in PPI network were CS, atp5f1a, sdha, ndufs1, uqcrc2, atp5f1c, atp5pb, vdac1, ndufv1 and uqcrc1.

### Selection of biomarker combinations for CRC with ***S. japonicum*** co-infected individuals

**Fig. 4:**
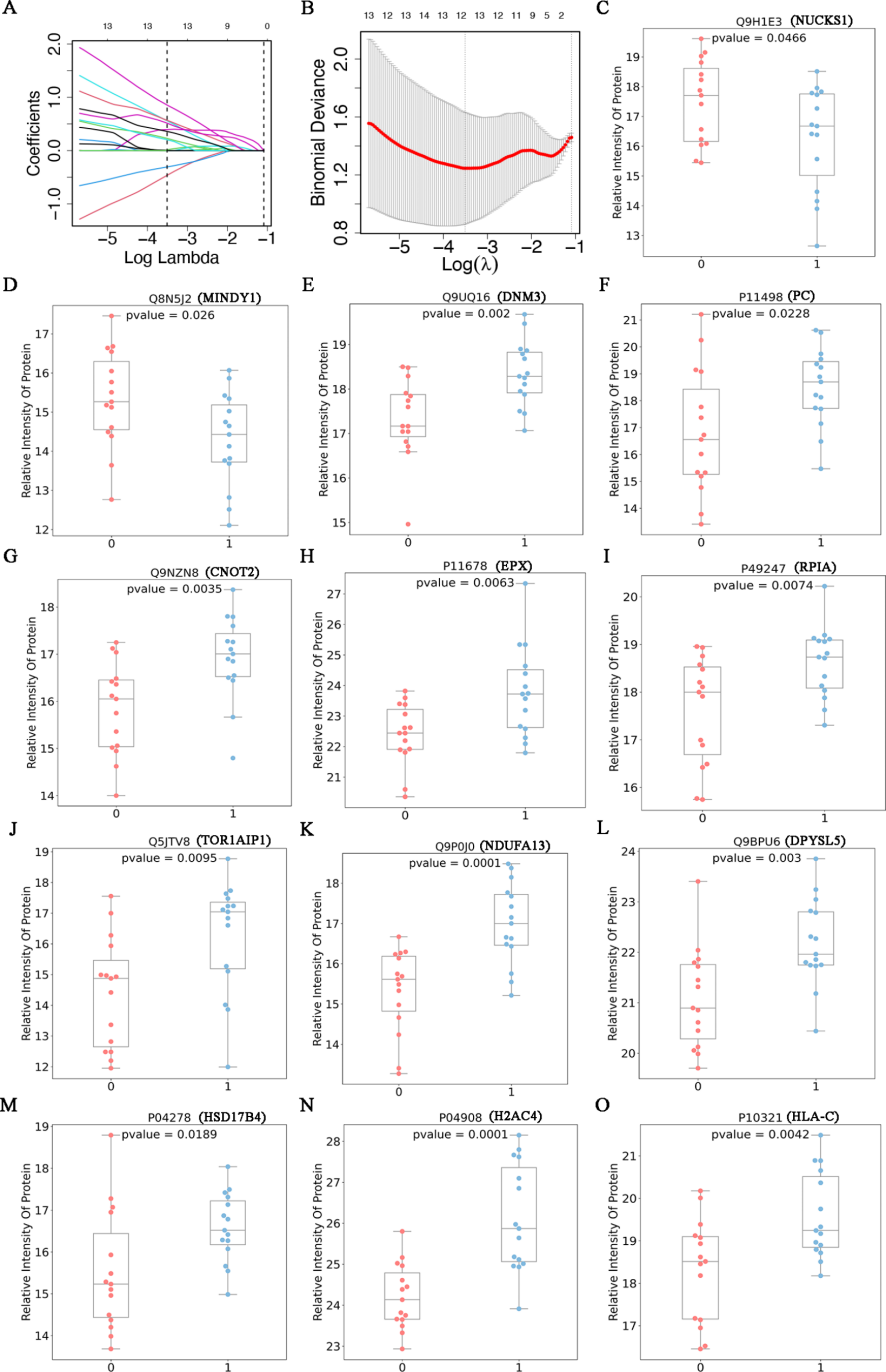
Screening genes that are correlated with the outcome variable from DEPs through LASSO regression analysis, providing a foundation for subsequent modeling. (**A**) Protein coefficient distribution plot. The different color lines represent the coefficient distribution curves of 13 proteins. The horizontal axis represents the Lambda value, the two dashed lines in the Fig indicate the variable and Lambda corresponding to the minimum fitting error on the left, and one standard deviation of the minimum fitting error of Lambda on the right. The y-axis represents the coefficients of the different variables. (**B**) Error Curve. The red line represents deviation curve of 13 proteins. The abscissa represents Lambda value. The ordinate represents the fitting error (**C**-**O**). LASSO regression evaluation results. The horizontal axis represents group information, and the vertical axis represents predicted values. The red box plot represents the group of CRC, while the blue box plot represents the group CRC combined with *S. japonicum* infection. The parameters that minimize the error were selected as the LASSO regression results for this analysis

The characteristic of LASSO regression is to perform variable selection and complexity adjustment while fitting the generalized linear model. It is mainly used for protein variable selection to screen out important proteins for subsequent modeling and analysis. Further LASSO regression analysis was performed on the DEPs to select genes that are associated with outcome variables, in order to provide a basis for subsequent modeling. A total of 13 proteins were selected for subsequent analysis, which are hsd17b4, h2ac4, hla-c, pc, epx, rpia, tor1aip1, mindy1, dpysl5, nucks1, cnot2, ndufa13 and dnm3. Figure 4 A shows the distribution of protein coefficients, Fig. 4B shows the error curve. In this analysis, the parameters that resulted in the smallest error were selected as the LASSO regression results. The proteins selected by LASSO can clearly distinguish individuals colorectal cancer with *S. japonicum* co-infection, as shown in Fig. 4C-O.

### Constructing a model for CRC complicated with ***S. japonicum*** co-infected

**Fig. 5:**
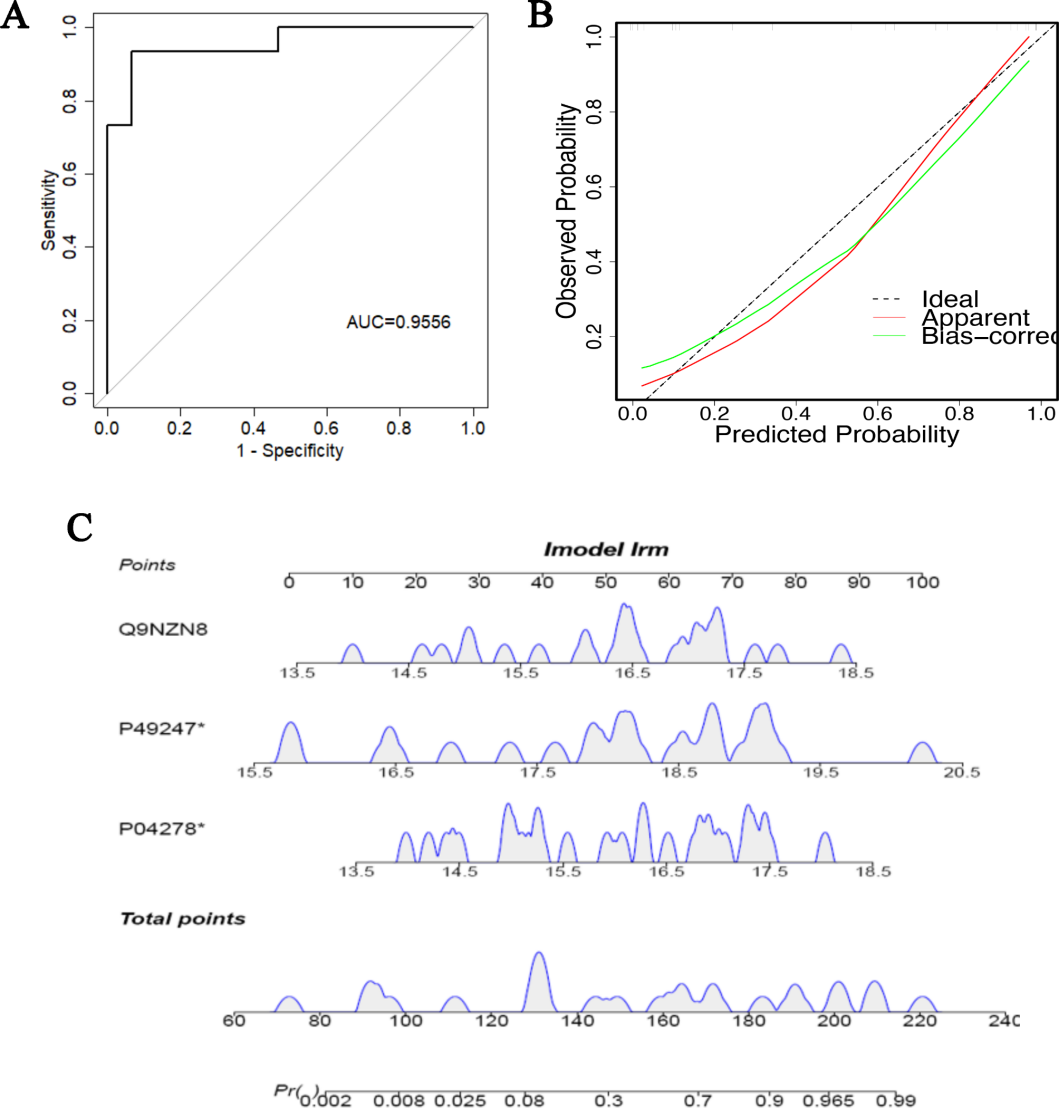
Construction of a visual logistic model of CRC with *S. japonicum* co-infected individuals. (**A**) Variable combination with best AUC value shown by logistic regression ROC curve was finally included in model analysis. (**B**) Calibration curve showed the model was predictive. (**C**) For each patient, three lines are drawn upward to determine the points received from the three predictors in the nomogram. The sum of these points is located on the “Total Points” axis. Then a line is drawn downward to determine the possibility of *S. japonicum* infection. AUC, area under curve; ROC, receiver operating characteristic curve

Using the screened important related proteins for modeling analysis, any number of combinations of important proteins will be selected. Through cross-validation analysis, the variable combination with the best AUC value will be selected as the final set of variables to be included in the model. The selected variables will be used to build the final model. Among the 13 key proteins obtained from LASSO analysis, hsd17b4 (p04278), rpia (p49247), and cnot2 (q9nzn8) were ultimately selected as the variable combination with the best area under the curve (AUC) value for the predictive model, and a clinical model was established based on this variable combination. As shown in Fig. 5A-B, the results of sensitivity and predicted probability analysis indicated that both sensitivity and predictive value of the model were effective. As shown in alignment diagram, total points from the sum of three proteins can determine the possibility of liver fibrosis (Fig. 5C).

## Discussion

Schistosomiasis is a parasitic disease that poses a serious threat to human health and economic development. It is a zoonotic disease that affects both humans and animals. The disease is caused by several species of blood flukes, including *Schistosoma mansoni*, *Schistosoma haematobium*, and *Schistosoma japonicum*. Schistosomiasis is prevalent in over 78 countries worldwide, and nearly 800 million people are at risk of infection [9]. Schistosomiasis infection can cause malnutrition and mechanical damage to tissues and organs in the human body, as well as harm the human body through the secretion of toxic and antigenic substances. This places a great burden on patients, their families, and society in terms of both disease and economic impact, and increases the burden of disease-adjusted life years for affected populations [11]. In China, the most prevalent type of schistosomiasis is *S.japonicum*. Although the infection rate of *S.japonicum* in China has significantly decreased, there are still a large number of patients with chronic schistosomiasis infection and late-stage schistosomiasis. The human portal vein-mesenteric vein system is the optimal environment for the survival of schistosomes. During the prolonged parasitic process, the large number of schistosome eggs produced can alter the intestinal environment, leading to chronic inflammatory reactions and thereby affecting the occurrence and development of tumors. An increasing amount of epidemiological and pathological evidence indicates that chronic infection with *S.japonicum* is related to the occurrence of various malignant tumors, especially colon cancer and liver cancer [4].

Colorectal cancer (CRC) is the third most common malignant tumor globally and the third leading cause of cancer-related deaths [12]. CRC mainly occurs in the sigmoid colon and rectum; the early clinical symptoms are often atypical and lack specificity. As the disease progresses, patients may experience abdominal pain, bloating, weight loss, changes in bowel habits, and rectal bleeding. At this point, the disease has often advanced to the middle or late stages. The pathogenesis of CRC is generally believed to be related to genetics, inflammatory bowel disease, high-fat dietary habits, excessive consumption of red meat, and other factors [13], however, the exact etiology and related mechanisms of the disease still need further investigation. Research has shown that about 17% of cancer cases can be attributed to chronic infections, and infection is considered one of the main factors in the development of cancer [14]. A large body of evidence indicates that the incidence of CRC in patients with *S.japonicum* infection is significantly higher than that in the normal population, with a three-fold increased risk of developing the disease compared to non-infected individuals [6]. In particular, patients with longer disease courses have an even higher probability of developing colorectal cancer than the normal population. The process of chronic inflammation in the intestines caused by *S.japonicum* infection leading to carcinogenesis may be one of the important mechanisms for the development of CRC after infection with this parasite [16]. Therefore, the International Agency for Research on Cancer (IARC) has classified *S.japonicum* as a Group 2B carcinogen, meaning it is possibly carcinogenic to humans [18]. *S. japonicum*-associated CRC has unique characteristics, including young age at diagnosis, male predominance, distal colonic location, multifocal distribution, and poor prognosis [4]. The causal relationship and benefit of screening and treatment for Schistosomiasis are debated and need further research.

Our research results will provide clues to the pathogenesis of CRC related to *S.japonicum* infection, and ultimately contribute to improving the diagnosis, prevention, and targeted treatment of early CRC combined with *S.japonicum* infection. Through proteomic techniques, proteins and post-translational modification sites can be identified or quantified on a large scale in biological samples, and valuable information and patterns can be extracted from high-throughput data analysis, providing clues and foundations for functional mechanism exploration. In our proteomic study, 115 differential proteins were identified in individuals with colon cancer combined with or without *S. japonicum* infection, with 110 proteins upregulated and 5 downregulated in colon cancer patients with *S.japonicum* infection compared to those with simple colon cancer. These differential proteins may be related to the pathogenesis of CRC with *S. japonicum* infection, including energy metabolism, mitochondrial function, and membrane transport. In addition, we also conducted functional and pathway analyses on the differential proteins, and found that these proteins are involved in multiple signaling pathways and biological processes, such as chemical carcinogenesis-reactive oxygen species, neutrophil extracellular trap formation, and necrosis signaling pathways. These findings provide important information and clues for revealing the relationship between *S.japonicum* infection and the pathogenesis of CRC.

In CRC combined with schistosoma infection, energy metabolism may play an important role. Some studies have shown that *S. mansoni* infection can affect the host’s energy metabolism and metabolic pathways, leading to metabolic disorders, which promotes mitochondrial fragmentation in the mouse liver [19], in addition, it also affects mitochondrial function and attenuates mitochondrial membrane potential, disrupts the mitochondrial dynamics of the mouse liver and induce mitochondrial fission [20]. Mitochondria play a critical role in energy synthesis and regulation in cells. Research have showed that infection with *S.japonicum* may affect host mitochondrial function, leading to mitochondrial respiratory chain complex dysregulation, increased oxidative stress within mitochondria, and damage to mitochondrial DNA. These changes may contribute to energy metabolism imbalance, increased cellular apoptosis, and ultimately promote the occurrence and development of CRC [21]. Studies have shown that infection with *S.japonicum* may lead to changes in the expression of transmembrane transport proteins on the cell membrane, affecting the transport and metabolism of substances. These changes may result in an imbalance of the intracellular and extracellular environment, thereby affecting cell growth and differentiation and increasing the risk of the occurrence and development of CRC [23]. The chemical carcinogenesis-reactive oxygen species signaling pathway plays an important role in patients with CRC and concurrent infection with *S.japonicum*, Tuffour et al. suggested that Schistosoma egg antigens could induce oxidative stress and reduce apoptosis in prostate cells, thereby stimulating tumor cell proliferation [25]. Soluble egg antigen (SEA) of *S. japonicum* has strong immunogenicity and can activate inflammatory cells such as macrophages, producing potential genotoxic mediators such as reactive oxygen species, reactive nitrogen species, and pro-inflammatory cytokines, leading to DNA damage, mutations, and dysregulation of oncogenes and tumor suppressor genes [26]. And bone marrow-derived mesenchymal stem cells (BM-MSCs) can also exert non-immunological functions, including promoting angiogenesis, tumor invasion, and metastasis, thereby favoring the progression of colorectal cancer [27]. Recently, a study found that the *S. japonicum* egg-derived protein SjE16.7 may induce the progression of CRC,it binds to the receptor for advanced glycation end products (RAGE) and activates the NF-κB signaling pathway, leading to an increase in reactive oxygen species, as well as pro-inflammatory cytokines IL-6 and TNF-α. This contributes to the stability of the tumor microenvironment and promotes tumor development [28]. As a whole, the carcinogenesis induced by *S. japonicum* may be related to chronic inflammation, schistosome toxins, immune regulation, and bacterial coinfection, in which chronic inflammation plays a critical role. After chronic infection with *S. japonicum*, it is a long-term inflammatory response, which is a process of continuous damage, repair, and proliferation [29]. Although no carcinogenic substance has been extracted from schistosome eggs, various inflammatory mediators are released during the inflammatory process, affecting genomic instability, causing the dysregulation of various oncogenes and tumor suppressor genes, thereby promoting tumor occurrence. Necroptosis, also known as programmed necrosis, is a form of programmed cell death that is thought to play a role in killing cells infected with pathogens or damaged cells during certain degenerative or inflammatory diseases. There is evidence to suggest that the transcriptional program activated downstream of RIPK3 is mainly responsible for driving the immunogenic role of necroptosis in environments ranging from infection to cancer transformation and autoimmunity. Additionally, some pathogens such as *Staphylococcus aureus*, *Streptococcus pneumoniae*, and *Listeria monocytogenes* can induce RIPK3-dependent programed cell death in macrophages, which may contribute to their immune escape [30].

Among the 13 key proteins obtained from LASSO analysis, hsd17b4, rpia and cnot2 were ultimately selected as the variable combination with the best AUC value for the predictive model, and a clinical model was established based on this variable combination. The hsd17b4 protein is a ketosteroid reductase involved in the metabolism of androgens and estrogens, and plays an important role in CRC [31]. Previous results have reported that hsd17b4 is a multiple-function enzyme involved in the progression of various cancers [32]. hsd17b4 is a potential biomarker for the diagnosis of prostate cancer (PCa), and studies implicate an increase in hsd17b4 expression related to poor prognosis for prostate cancer, upregulating hsd17b4 enhanced the malignant capacities of PCa cells, while hsd17b4 knockdown inhibited these capacities [33]. Our study showed that the expression level of hsd17b4 in colorectal cancer samples with *S.japonicum* infection was higher than that in pure colorectal cancer samples, indicating a poor prognosis. The pentose phosphate pathway (PPP) is crucial for the survival and proliferation of cancer cells, and ribose-5-phosphate isomerase A (RPIA) is an important component of the PPP, regulating cancer cell growth and tumor development, RPIA was known for its role in oncogenic signaling [34]. RPIA is a phosphoribose isomerase that participates in the reaction of the pentose phosphate pathway in ribose metabolism. Recent studies have shown that RPIA is upregulated in various tumors and plays a role in tumorigenesis and progression. RPIA fulfills the biosynthetic requirements of cancer cells, as evidenced by deficient DNA synthesis in its absence, and by its overexpression in colorectal cancer, liver cancer, and pancreatic cancer [35]. Besides, RPIA knockdown slightly suppresses the colony formation of iKras p53^L/+^ tumor cells in high glucose, the inhibitory effect is significantly enhanced when cells were cultured under low glucose condition [38]. These observations suggest that RPIA might be a critical node connecting glucose availability with metabolic needs in malignancy. Therefore, the upregulation of RPIA in the group of colorectal cancer combined with *S. japonicum* infection may be related to its poor prognosis. The cnot2 protein is a component of the CCR4-Not transcription complex, involved in the regulation of mRNA metabolism and degradation [39]. Recent studies showed that inhibition of cnot2 in human cancer cells inhibits cancer cell proliferation and angiogenesis through VEGF signaling in cancer cells, suggesting that cnot2 acts as an oncogene [40]. The research results indicate that cnot2 plays a tumor-promoting role in colon cancer.

We acknowledge some limitations of our study. First, our study was based on proteomic analysis of paraffin-embedded samples and further direct indicators, such as serological indicators, are needed for further validation. In this regard, we have been collecting relevant serum samples. Second, our study did not provide further validation studies, which are particularly important and necessary. In order to strengthen and promote the application value of predictive indicators for the occurrence of colorectal cancer combined with *S. japonicum* infection, verification studies need to be conducted in a larger population, including normal population, individuals with *S. japonicum* infection alone, and individuals with colorectal cancer combined with *S. japonicum* infection. Third, the sample size of this study was relatively small. In the future, we will create conditions to supplement appropriate samples to further improve the experimental results and analysis. However, despite these limitations, the results of our bioinformatic analysis provide valuable clues for future mechanistic studies on colorectal cancer combined with *S. japonicum* infection.

## Materials and methods

### Study design and sample collection

The study was conducted at the Affiliated Hospital of Jiujiang University, it’s near Poyang Lake, Jiangxi Province, China. Patients who received their first rectal cancer resection in the Department of General Surgery from January 2016 to December 2021 were retrospectively collected and divided into colorectal cancer group 0 (CRC) and colorectal cancer combined with *S. japonicum* infection infection group 1 (CRC-Sj). Two senior professional physicians of the hospital took a double-blind method to read the film and make the diagnosis. In case of disagreement, the diagnosis made by two physicians after discussion. The diagnostic criteria for CRC-Sj group was as follows: In colorectal cancer patients with a history of *S. japonicum* infection, postoperative pathology shows *S. japonicum* egg deposition in tumors and surrounding tissues. The patients who agreed to participate in this study were included. According to the inclusion and exclusion criteria, 15 patients with simple colorectal cancer and 15 colorectal cancer patients with *S. japonicum* infection were included in this study. The paraffin sections and clinical information of the patients were collected. The basic information of the patients is shown in Table 1.

### Sample preparation

The formalin-fixed and parrffin-embedded (FFPE) sample was cut into 20 μm thin slices with a microtome, and 10 pieces were cut into the same 1.5mL EP tube. (Note: If there is no special requirement, the section with the largest cross section should be cut to ensure that the length and width of the cross section are ≥ 3 mm. When dealing with different FFPE samples, try to keep the same position of the cross section for cutting, so as to maximize the consistency of the experiment). The reaction solution (1%SDC/100 mM Tris-HCL pH = 8.5/10 mM TCEP/40 mM CAA) was added to the sample and incubated at 60℃ for 1 h for protein denaturation, reduction and alkylation in one step. After dilution with equal volume of ultra-pure water, trypsin was added according to the mass ratio of enzyme to protein of 1:50, and incubated at 37 ℃ and shook overnight for enzyme digestion. Following day, TFA was added to terminate enzyme digestion, and 16,000 g of supernatant was centrifuged for desalting self-made SDB column. After draining, freeze storage at -20 ℃ for use.

### Mass spectrometry detection

Mass spectrometry is a powerful analytical technique used to determine the molecular mass, composition, and structure of a sample. The mass spectrometry detection of peptide samples was performed using a Q Exactive HF-X mass spectrometer coupled with an EASY-nLC 1200 liquid chromatography system. The samples were dissolved in loading buffer and automatically injected into an analytical column (75 μm*25 cm, C18, 2 μm, 100Å) for separation. A 100-min gradient was established using two mobile phases (mobile phase A: 0.1% formic acid and mobile phase B: 0.1% formic acid, 80% ACN) at a flow rate of 300 nL/min. In the data-dependent acquisition (DDA) mode, each scan cycle consisted of a full MS scan (R = 60 K, AGC 1e6, max IT = 20 ms, scan range = 350–1800 m/z), followed by 25 MS/MS scans (R = 15 K, AGC = 2e5, max IT = 50 ms). The HCD collision energy was set to 28, and the quadrupole isolation window was set to 1.6 Da. The dynamic exclusion time for ion re-acquisition was set to 35s. In the data-independent acquisition (DIA) mode, each scan cycle consisted of one MS1 scan (scan range 350-1,250 m/z, resolution 60 K, AGC 3e6, max. IT = 30 ms) and 40 variable window MS2 scans (resolution 15 K, AGC 1e6, max. IT 50 ms). The collision energy was set to 28.

### Data processing

First, the DDA raw data files were searched against the Human_Salmonella protein reference database (May 10, 2020, containing 79,618 protein sequences) using the MSFraggersoftware to obtain protein identification results [41]. The search parameters included variable modifications, enzymatic cleavage, and matching tolerance. Then, a spectral library was constructed based on the DDA identification results for subsequent DIA data analysis. Next, the DIA raw data files were analyzed using the DIA-NN software, which extracted protein quantification information using the previously constructed spectral library, and filtered the results at a 1% FDR for both precursor and protein levels. The filtered protein quantification information was used for subsequent analyses.

The data was filtered based on the actual number of values exceeding the median in each group, followed by log transformation and normalization (subtracting the median). Finally, data imputation was performed using the random small value method, and the imputed data was used for subsequent analysis. Categorical data was analyzed using the chi-square test and Fisher’s exact test, normally distributed continuous data was analyzed using the t-test, and non-normally distributed continuous data was analyzed using the rank-sum test.

### Differentially expressed proteins screening

Database retrieval of the raw mass spectrometry data can yield the detection signal intensity of each peptide, which can be used to calculate the corresponding protein quantification information. Quantitative comparisons can be made between the same protein in different samples. In this study, a fold change of 1.2 was selected, with an upper bound of 2 and a lower bound of 1/2 and p < 0.05 were identified as differentially expressed proteins (DEPs). The numerator group is group 1, and the denominator group is group 0. Quantitative analysis of differential proteins includes correlation analysis, quantitative heat map generation, hierarchical clustering analysis, and principal component analysis (PCA), which reflect the role of differential proteins between the two groups.

### Functional enrichment analysis

The human protein database was used as the annotation reference database. The R package clusterProfiler [42] was used to annotate and perform enrichment analysis on both identified proteins and differentially expressed proteins. The Gene Ontology (GO) database (http://geneontology.org/) was used to annotate proteins based on their Biological Process, Molecular Function, and Cellular Component. The Disease Ontology (DO) database (https://disease-ontology.org/about/) was used to perform enrichment analysis based on the human database [43]. The Kyoto Encyclopedia of Genes and Genomes (KEGG) database (https://www.kegg.jp/kegg/) was used to perform pathway analysis and the analysis was carried out using clusterProfiler [45]. The enrichment results were ranked based on protein values, and the top 20 enriched entries were selected for graphical representation. Enriched metabolic pathways were selected from the KEGG results and were drawn using the KEGG API. Differentially expressed proteins in the pathway were marked in red or blue, indicating up- or down-regulation, respectively, in our experiment.

### Protein–protein interaction network construction

The STRING database (https://string-db.org/) is a data system used to retrieve information on known and predicted protein-protein interactions [48]. In addition to experimental data-supported protein interaction information, this database contains protein interaction information obtained through text mining of PubMed abstracts, as well as protein-protein interactions predicted using bioinformatics methods. The bioinformatics methods used to predict protein-protein interactions include chromosome proximity, gene fusion, systematic evolution of ligands by exponential enrichment (SELEX), and co-expression analysis of genes. This study analyzes the protein-protein interaction network of differentially expressed proteins in two comparison groups based on the STRING database.

### Machine learning and model construction

The LASSO regression is a method that simultaneously performs variable selection and complexity adjustment while fitting a generalized linear model. It is mainly used for protein variable selection to identify important proteins for subsequent modeling and analysis. Our study utilized LASSO regression to select important proteins, which were subsequently used for modeling and analysis. And the selected proteins were considered as important candidate genes for distinguishing colorectal cancer combined with *S. japonicum* infection. We performed cross-validation analysis on various combinations of the selected proteins to identify the best-performing variable combination based on the area under the curve (AUC) value, which was subsequently included in the final model. The selected variables were used to build the final model.

## Data Availability

The datasets generated and analysed during the current study are not publicly available, but are available from the corresponding author on reasonable request. The datasets used and analyzed during the current study are as following: UniProt (https://www.uniprot.org/), Gene Ontology (http://geneontology.org/), KEGG (https://www.kegg.jp/kegg/), STRING (https://string-db.org/), Dsease-Otology (https://disease-ontology.org/about/).
